# Unilateral biportal endoscopic decompression for acute spontaneous spinal epidural hematoma at the cervicothoracic junction: a case report and literature review

**DOI:** 10.3389/fsurg.2026.1756449

**Published:** 2026-06-25

**Authors:** Longyao Cao, Hui Lu, Dongjie Zhang

**Affiliations:** Department of Spinal Surgery, Bishan Hospital of Chongqing Medical University, Chongqing, China

**Keywords:** case report, literature review, minimally invasive spine surgery, spinal cord compression, spontaneous spinal epidural hematoma, surgical decompression, unilateral biportal endoscopy

## Abstract

**Background:**

Spontaneous spinal epidural hematoma (SSEH) represents a rare neurosurgical emergency. While conventional open surgical decompression remains the standard of care, it necessitates substantial tissue dissection and is associated with prolonged recovery periods.

**Methods:**

The patient underwent enhanced magnetic resonance imaging (MRI) confirming SSEH at C7-T2. Surgical decompression and hematoma evacuation were performed using a unilateral biportal endoscopy (UBE) technique under general anesthesia.

**Results:**

UBE was completed successfully in 90 min with an estimated blood loss of 50 mL. The patient demonstrated significant neurological improvement, achieving independent ambulation with a walker by postoperative day 3. At the 6-month follow-up, she attained ASIA grade E with only minimal residual increase in lower limb muscle tone.

**Conclusions:**

UBE offers a viable minimally invasive alternative to open surgery for SSEH evacuation. This technique provides excellent visualization and precise decompression while preserving spinal stability, resulting in reduced surgical trauma and accelerated neurological recovery.

## 1 Introduction

Spontaneous spinal epidural hemorrhage (SSEH) is a rare condition, with an estimated occurrence rate of 0.1 per 100,000 individuals per year (1). The etiology of the hemorrhage has been debated for decades; however, it is generally accepted that rupture of the vertebral venous plexus appears to be the most likely source of bleeding (2–4). Although their exact significance remains unclear, risk factors for SSEH include anticoagulant therapy, coagulation disorders, arteriovenous malformations, hypertension, physical exertion, and increased abdominal or thoracic pressure (5). The hallmark symptom of SSEH is the acute onset of severe, occasionally radiating back or neck pain, followed by rapidly evolving signs and symptoms of nerve root or spinal cord compression. This clinical presentation often mimics more common pathologies, such as acute ischemic stroke (AIS), which can lead to delays in treatment. Prompt recognition of potential SSEH and performing magnetic resonance imaging (MRI) are crucial to enable urgent intervention (6). SSEH can potentially affect any level of the spinal canal; however, it predominantly occurs in the cervicothoracic (C5-T2) and thoracolumbar (T10-L2) regions. Patients with SSEH presenting with neurological deficits are subjected to spinal decompression and hematoma evacuation (7, 8). Nevertheless, open surgery is often associated with significant trauma and prolonged recovery periods. In recent years, with advancements in minimally invasive surgical techniques, the unilateral biportal endoscopic approach has gained increasing favor among spine surgeons. Compared with traditional open surgery, unilateral biportal endoscopy (UBE) offers advantages such as minimal anatomical disruption, reduced postoperative pain, and shorter recovery time in the treatment of lumbar disc herniation or lumbar spinal stenosis (9, 10). However, the application of UBE for the treatment of SSEH has rarely been reported. This article aims to present a case of SSEH in which the patient achieved significant symptomatic relief following UBE intervention.

## 2 Case presentation and surgical technique

### 2.1 Patient information

A 67-year-old female patient presented with a chief complaint of sudden-onset sensory and motor disturbances in the trunk and both lower extremities for 4 h. Physical examination revealed decreased pain and touch sensation below the nipple plane, muscle strength of key muscles in both upper extremities graded as 4, muscle strength in both lower extremities graded as 0, and bilateral Babinski signs (+). The patient specifically denied any history of antiplatelet or anticoagulant medication use prior to admission, and the patient had no identifiable coagulation disorders, as confirmed by normal preoperative coagulation studies (prothrombin time 11.3 s, activated partial thromboplastin time 25.8 s, international normalized ratio 1.04). After admission, the patient underwent detailed imaging examinations. Contrast-enhanced MRI revealed a space-occupying lesion located dorsally in the dural sac at the C7-T2 level, causing spinal canal stenosis and compression of the dural sac (Figure 1). The lesion appeared smooth, spindle-shaped, and homogeneously slightly hypointense on T1-weighted images. Based on her symptoms, physical examination, and imaging findings, the space-occupying lesion in the dorsal dura at the C7-T2 level was considered the cause of her symptoms. After obtaining informed consent, decompression and resection of the epidural space-occupying lesion were performed using the UBE technique.

**Figure 1:**
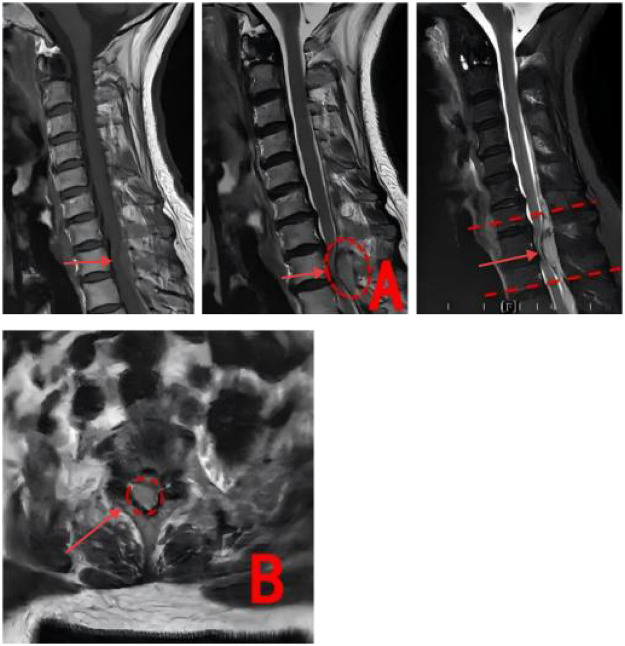
Preoperative spinal MRI. **(A)** Sagittal T1-weighted images at the C7–T2 level showed slightly hypointense signals, T2-weighted images revealed hypointense signals, and fat-suppressed sequences demonstrated slightly hyperintense epidural compression dorsal to the dural sac, resulting in spinal canal stenosis. Red arrows indicate the extent of the epidural hematoma compressing the dural sac. **(B)** Axial T2-weighted images at the C7–T2 level revealed spinal canal stenosis, an epidural mass, anterior displacement and compression of the dural sac. Red arrows indicate the extent of the epidural hematoma compressing the dural sac.

### 2.2 Surgical technique

Under general anesthesia, the patient was placed in a prone position on a radiolucent operating table. The responsible spinal level was identified using C-arm fluoroscopy. Two transverse skin incisions were made along the right paramedian line, centered over the T1-T2 intervertebral space, with one incision proximal and the other distal to the target level. The proximal incision served as the working portal, and the distal incision served as the viewing portal. The skin and fascia were incised sequentially. Endoscopic instruments were placed over the lamina and interlaminar space. The muscular tissue overlying the lamina and interlaminar space was dissected, and the interlaminar ligamentum flavum was resected. The right lamina of T1, the superior margin of the partial right lamina of T2, and the inferior margin of the partial right lamina of C7 were removed using a high-speed drill and pituitary rongeurs under endoscopy. The epidural space was filled with hematoma, causing compression of the dural sac. The hematoma was carefully dissected and removed with forceps (Figure 2). Under endoscopy, the restoration of rhythmic pulsation of the dura mater was considered an indicator of adequate decompression. After meticulous hemostasis was achieved, the working cannula and endoscope were withdrawn. Finally, the skin wounds were closed with sutures.

**Figure 2:**
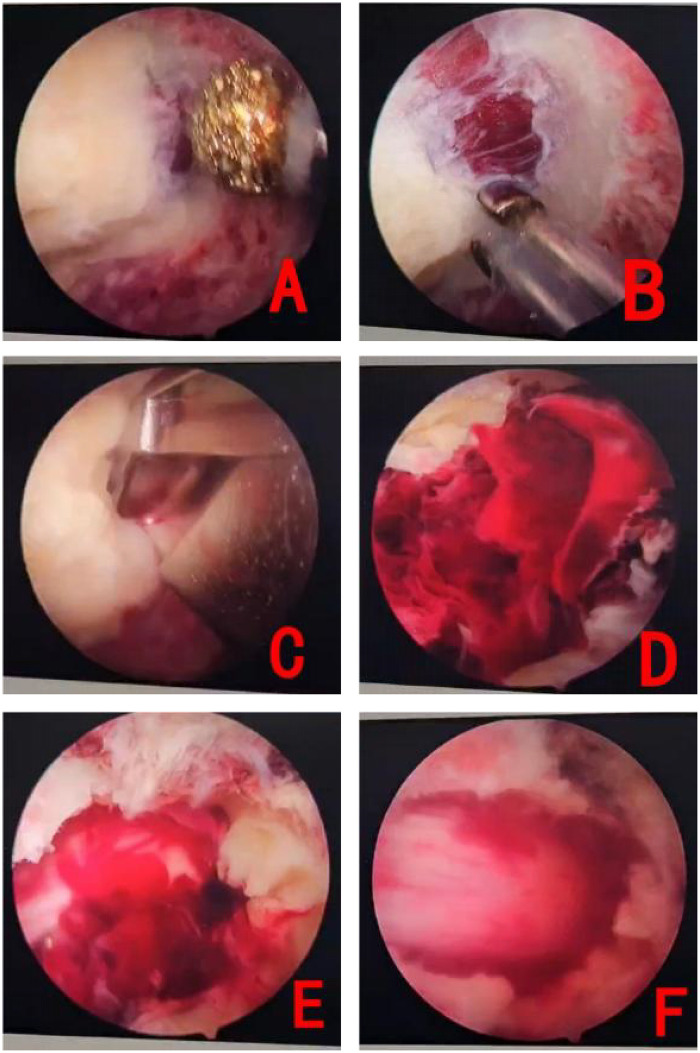
Intraoperative exploration and decompression. **(A)** Grinding of the right hemilamina of T1. **(B)** Exposure of the C7 and T2 ligamentum flavum insertion points. **(C)** Removal of the ligamentum flavum. **(D)** Exposure and removal of dorsal compressive material on the dura mater. **(E)** Decompression of the dural sac. **(F)** The hematoma was evacuated, and no compressive material remained dorsal to the dural sac.

### 2.3 Postoperative imaging examinations and follow-up

Twenty-four hours postoperatively, the patient's muscle strength in all four limbs gradually recovered and showed significant improvement compared to the preoperative status (upper limbs: Grade 5, lower limbs: Grade 4). Postoperative imaging confirmed complete resection of the lesion located dorsal to the spinal dura within the spinal canal (Figure 3). Upon inquiry regarding the recovery process, the patient was able to ambulate upright with the assistance of a walker, although mild weakness of the lower limbs was still perceived during standing and walking. Apart from mild lower limb muscle weakness, the patient reported no other symptoms. At the 6-month postoperative follow-up, the ASIA Impairment Scale grade had improved to E, with residual partial increase in lower limb muscle tone.

**Figure 3:**
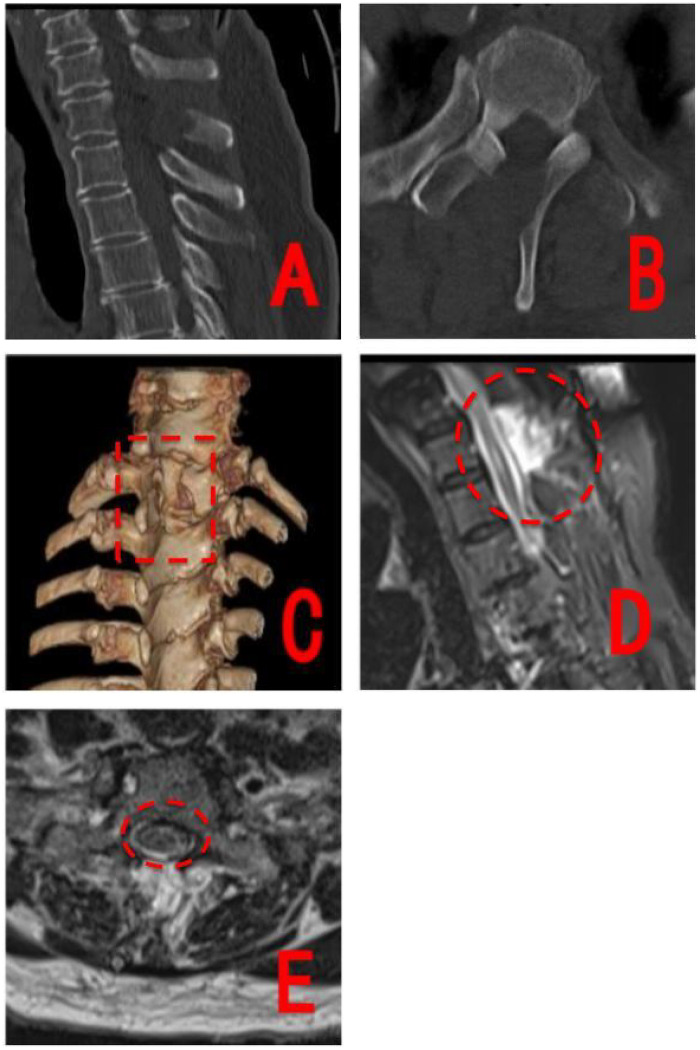
Postoperative CT and MRI of the cervicothoracic spine. **(A)** Sagittal CT image demonstrates the absence of an epidural mass at the C7-T2 level, with evidence of partial laminectomy. **(B)** Axial CT image reveals adequate spinal canal decompression without residual epidural compression. **(C)** Three-dimensional CT reconstruction illustrates the extent of the laminectomy. **(D)** Sagittal fat-suppressed T2-weighted MRI confirms resolution of dorsal compression and shows postoperative changes with a small residual hematoma. **(E)** Axial T2-weighted MRI at the C7-T2 level indicates no residual mass, sufficient spinal canal decompression, and a small postoperative hematoma.

## 3 Discussion

To date, the literature on endoscopic treatment of spontaneous spinal epidural hematoma (SSEH) remains limited, reflecting the rarity of this condition. Kim and Jung (11) described biportal endoscopic spine surgery for a multi-level spontaneous lumbar epidural hematoma, achieving favorable neurological recovery with minimal invasiveness. Zhuang et al. (12) reported successful unilateral biportal endoscopic (UBE) evacuation of a post-traumatic epidural hematoma at the T12/L1 level with good postoperative outcomes. To the best of our knowledge, the present case is the first reported application of UBE for acute SSEH at the cervicothoracic junction (C7-T2). Table 1 summarizes the key characteristics of these previously reported endoscopic SSEH cases.

**Table 1:** Summary of previously reported endoscopic cases of spontaneous spinal epidural hematoma.

Author (Year)	Hematoma Location	Endoscopic Technique	Clinical Outcome
Kim and Jung (11)	Lumbar (multi-level)	UBE	Favorable neurological recovery
Zhuang et al. (12)	T12/L1 (post-traumatic)	UBE	Good postoperative outcome

UBE, unilateral biportal endoscopy.

Patients with SSEH are typically in their 40s or 50s (13, 14), with a slight male predominance (male-to-female ratio 1.4:1) (15). The estimated annual incidence is 0.1 per 100,000 patients (16). SSEH usually presents with sudden onset of severe neck or back pain, which may radiate to the extremities, followed by a spectrum of neurological symptoms ranging from nerve root irritation to complete neurological dysfunction (13, 14, 17–20). Lower motor neuron signs, including hyporeflexia and flaccid paralysis, are characteristic manifestations (17). A time delay between the onset of back pain and neurological deterioration may be observed, with reported intervals ranging from hours to days or even months after initial symptom presentation (13, 17, 19). Early suspicion and diagnostic imaging are crucial, as SSEH can lead to profound and permanent neurological deficits, including persistent paralysis or even death.

The formation of SSEH may be associated with various pathological factors. In the literature, 40%–60% of cases indicate no identifiable hemorrhagic risk factors for SSEH development (21, 22). Trauma and surgery are known risk factors for the occurrence of epidural hematoma (17). As reported in the literature (18, 23, 24), the use of anticoagulants appears to be a risk factor for SSEH, whereas platelet inhibitors are not. Hypertension was observed in 23.7% of patients, which is comparable to data from the general population in this age group (25). A meta-analysis indicated that hypertensive patients do not exhibit an increased risk of developing SSEH (18), which aligns well with the hypothesis that SSEH originates from venous sources. The source of bleeding has been a subject of considerable debate, with Batson's epidural venous plexus (14, 26, 27), epidural arteries (28), and vascular malformations all proposed as potential bleeding sites (22). When patients present with significant progressive neurological deterioration, an arterial source is more likely (14). However, most authors support a venous origin of bleeding, as anatomical evidence and the occasionally slow progression of symptoms favor venous sources (13, 14, 17, 29). Furthermore, no known arterial structures within the spinal canal can account for the segmental distribution and localization of SSEH observed to date (30).

The vertebral internal epidural venous plexus, which drains the abdomen and thorax, is a low-pressure, valveless system that may rupture when pressure increases due to Valsalva maneuver (19). Compared to the ventral compartment, the dorsal epidural space is more frequently affected by SSEH (14, 18, 19). This may be attributed to the fact that the ventral epidural veins are partially situated beneath the posterior longitudinal ligament, thereby receiving greater structural support, while the dorsal epidural venous plexus is also larger in scale than its ventral counterpart (17, 18). Additionally, there may exist a “region of weaker resistance” that is more prone to rupture in response to minor fluctuations in venous pressure (17). The predilection of SSEH for middle-aged individuals can be explained not only by alterations in vascular composition but also by the cumulative effects of gravity leading to increased venous dilation (18). Other researchers have proposed that regions of the spine with greater mobility exert increased tension on the epidural veins (19), which may account for the frequent occurrence of SSEH in the cervicothoracic or thoracolumbar regions (14, 17–19).

Early surgical spinal cord decompression and drainage are required for SSEH to facilitate the potential for neurological recovery. To enable rapid diagnosis, prompt recognition of symptoms is crucial for obtaining MRI expediently, which is the imaging modality of choice for SSEH (31). Compared to the spinal cord within 24 h of onset, the hematoma appears isointense on T1-weighted images and hyperintense on T2-weighted images. After 24 h, the hematoma typically appears hyperintense on both T1-weighted and T2-weighted images (15, 17, 19). Chronic hematomas exhibit hypointensity on both T1-weighted and T2-weighted images (17). Fat-suppressed images can be used to differentiate hematomas from epidural fat. Occasionally, when contrast agents are administered, active bleeding within the hematoma may demonstrate a centrally enhanced area (20).

Once diagnosed, emergency surgical intervention is required (32). Some studies suggest that to achieve optimal neurological improvement, patients should undergo surgical decompression within 12 to 48 h after symptom onset (14, 19, 20). There is a consensus that urgent, or at least emergent, surgical intervention is necessary. Many authors recommend decompression within 24–36 h for complete injuries and within 48 h for incomplete injuries (13, 19). However, the patient's neurological status prior to surgical intervention is a more profound determinant of long-term outcomes (32). This represents the most significant prognostic indicator (15, 18).

Compared to the cervical region, patients with hematomas located in the thoracic or thoracolumbar junction regions exhibited poorer postoperative outcomes (32). This relationship is attributed to the narrower spinal canal in the thoracic region, the relatively smaller diameter of associated blood vessels, and the poorer collateral circulation, which collectively increase the risk of spinal cord ischemic injury in the presence of a compressive hematoma (18). Patients who developed more severe symptoms within a shorter time frame tended to have larger hematomas, particularly when four or more spinal segments were involved (32). The underlying rationale is that an increased number of affected segments is directly associated with more extensive bleeding, thereby leading to a worse prognosis.

The primary treatment options for SSEH typically involve hemilaminectomy or laminectomy followed by irrigation and debridement (13, 19, 20). If a significant coagulopathy is identified as the underlying cause, it should be corrected prior to surgical intervention (15). For patients managed non-operatively, serial neurological examinations are required during strict bed rest (20). However, outcomes are generally poor without surgical intervention (33). Therefore, non-surgical management is reserved only for patients who are not suitable candidates for surgery or those who remain asymptomatic (20, 32). Both laminectomy and hemilaminectomy have been reported to be equally effective in evacuating hematomas, with no significant risk of spinal instability observed even when the lesion spans up to five spinal levels (8, 34, 35). Nevertheless, previous studies have indicated that patients subjected to hemilaminectomy are more likely to achieve better postoperative outcomes compared to those undergoing laminectomy (36). The etiology of neurological deficits may involve not only mechanical compression but also subsequent inflammatory responses. Consequently, surgical intervention remains necessary even in patients presenting with ASIA A deficits or delayed symptom onset (13, 32). In our case, the patient received emergency surgical intervention, which resulted in a favorable outcome — the preoperative ASIA A deficit improved to ASIA E status postoperatively.

The cervicothoracic junction (CTJ, C7–T2) presents unique anatomical complexities that increase the risk of conventional open surgery. An et al. demonstrated that the cross-sectional area ratio of the spinal cord to the spinal canal decreases from 1:2.3 at C6 to 1:4 at T1, indicating a progressively narrower spinal canal relative to the cord in the lower cervical and upper thoracic region (37). This anatomical feature leaves less margin for error during instrument manipulation and increases the risk of iatrogenic cord injury. The same authors further reported that the lamina is thinnest at C7, which poses a risk of inadvertent dural or cord injury during drilling or Kerrison punch insertion (37). Moreover, the orientation of the lamina shifts from the more horizontal cervical pattern to the steeper, overlapping “shingled” thoracic configuration. This abrupt change makes interlaminar access more challenging and increases the risk of incomplete decompression or unintended injury if the instrument trajectory is not carefully adjusted. Additionally, the CTJ is anatomically adjacent to the thoracic duct, stellate ganglion, recurrent laryngeal nerve, and dome of the pleura. This proximity carries higher risks of unintended injury during anterior or extensile posterior approaches, particularly in open procedures that require wider exposure (38). Finally, the CTJ is a transitional zone between the mobile lordotic cervical spine and the rigid kyphotic thoracic spine, creating a region of concentrated mechanical stress. Conventional laminectomy at this level disrupts the posterior tension band and has been associated with an increased risk of post-laminectomy kyphosis and instrumentation failure (38).

In recent years, unilateral biportal endoscopy (UBE) has become an indispensable technique for treating degenerative spinal diseases. The small incisions minimize iatrogenic trauma, alleviate postoperative pain, and accelerate recovery (39–41). The clear endoscopic visualization enables precise localization and dissection of pathological tissues while protecting neural structures (42). Furthermore, the posterior approach in UBE is relatively straightforward. Importantly, its gentle learning curve allows more surgeons to master this technique. The UBE approach addresses the aforementioned anatomical challenges through several mechanisms. First, UBE preserves the posterior tension band by maintaining the integrity of the interspinous ligament and contralateral paraspinal muscles, thereby significantly reducing the risk of iatrogenic kyphotic deformity. In thoracic ossification of the ligamentum flavum, full-endoscopic decompression has been shown to cause less damage to the posterior ligament complex, with no cases requiring secondary internal fixation (41, 43). Second, the high-definition endoscopic view with continuous saline irrigation provides a clear, blood-free surgical field, which is particularly critical in the CTJ where the epidural venous plexus is prominent. This allows precise identification of the C7 lamina, T1 lamina, and the interlaminar space, minimizing the risk of wrong-level surgery (44). Third, UBE enables targeted unilateral hemilaminectomy without requiring complete exposure of the spinal canal. Given that the C7 lamina is the thinnest, the endoscopic approach allows controlled, layer-by-layer bone removal under direct visualization, avoiding the sudden loss of resistance that can occur with conventional Kerrison punches. Fourth, the angled endoscope (typically 30°) allows visualization across the midline, enabling contralateral decompression without stripping contralateral muscle attachments. This protects the contralateral facet joints and spinous process–ligament complex, reducing the risk of postoperative instability that would otherwise necessitate instrumented fusion. Fifth, because UBE operates within a narrow working corridor under continuous visualization and avoids wide tissue dissection, the risks to the thoracic duct, stellate ganglion, and pleura are considerably lower than in open procedures. This minimally invasive dissection is particularly beneficial in the CTJ region, where these structures lie in close anatomic proximity.

Unlike the microscope-assisted tubular approach, in which the endoscope and instruments share a single portal, leading to instrument crowding and reduced maneuverability, UBE features separate viewing and working portals. This separation allows the surgeon to simultaneously visualize the surgical field through one portal while manipulating instruments through the other, providing true “triangulation.” A recent systematic review and meta-analysis comparing the biportal endoscopic vs. tubular approach for lumbar degenerative disease (45) confirmed that UBE offers comparable safety with modest advantages in selected perioperative outcomes. For fragile intraspinal hematoma evacuation, this triangulation enables the surgeon to maintain constant visual confirmation of the hematoma-dural interface while the working instrument approaches from a different trajectory, minimizing the risk of inadvertent dural tearing or cord compression during clot removal. The continuous saline irrigation system in UBE provides a clear, blood-free surgical field, which is particularly critical when evacuating a fragile hematoma where bleeding points may be obscured by residual clot. In the microscope-assisted tubular approach, the tubular retractor maintains an air medium with intermittent irrigation, which can be hampered by recurrent bleeding that requires frequent instrument withdrawal and reinsertion, prolonging operative time and increasing risk. The fragile nature of an acute hematoma makes it prone to fragmentation during suction aspiration. UBE's independent irrigation and suction channels allow precise, low-pressure suction directly targeted at the clot under continuous visualization, reducing the risk of fragment dispersion into adjacent spinal canal spaces. The meta-analysis demonstrated that UBE was associated with a lower complication rate (10.73% vs. 15.94%; *P* < 0.001) and significantly lower early postoperative back pain scores compared to the tubular approach (45).

Despite the advantages of UBE in clinical practice, it still faces several challenges. Maintaining clear visualization in the surgical field is often compromised by limited anatomical space and intraoperative bleeding. The presence of intraspinal blood clots can obscure the surgical view and increase the risk of iatrogenic injury, necessitating the use of precise instrumentation. Coordination between endoscopic visualization and instrument manipulation is essential to prevent instrument–canal conflicts. Furthermore, the applicability of UBE in extensive pathologies involving multiple spinal segments remains uncertain due to technical limitations and a lack of robust evidence. Potential complications, including dural tears, nerve injury, or postoperative hematoma, require prompt management to mitigate associated risks. Postoperative care should focus on inflammation control through the use of anti-inflammatory medications and physical therapy, as well as adhesion prevention to optimize recovery.

A critical safety consideration during endoscopic spine surgery at the cervicothoracic junction is the management of irrigation pressure, as excessive pressure can potentially lead to elevated intracranial pressure (ICP) or spinal cord compromise. In the present case, we utilized gravity-assisted irrigation rather than a mechanical pump system. The saline bag was positioned approximately 60–70 cm above the surgical field, delivering an estimated irrigation pressure of 40–50 mmHg. This approach was deliberately chosen over higher pump pressures to minimize the risk of increased ICP or spinal cord injury. The outflow portal was maintained patent throughout the procedure via a fascial incision perpendicular to the skin incision, allowing continuous fluid egress. No clinical signs suggestive of increased ICP, such as headache, altered mental status, seizure, or autonomic dysreflexia, were observed during or after the procedure. However, direct ICP monitoring was not performed, which we acknowledge as a limitation.

A growing body of literature has established that continuous saline irrigation during endoscopic spine surgery can raise ICP to potentially critical levels, particularly when drainage channels are occluded. Öztürk et al. (46), using eight fresh cadavers, demonstrated that cranial pressure increases were immediate and significant after irrigation initiation, with the highest pressure values observed at 195 mmHg pump pressure; the average pressure difference across all measured points was 29.74 mmHg. Farshad et al. (47) developed an experimental cadaver model confirming that headache, seizure, and autonomic dysreflexia are possible irrigation-related complications following full-endoscopic lumbar discectomy, and that pressure elevation through fluid irrigation may contribute to these adverse events. Importantly, that study also demonstrated that pressure increase could be detected sensitively by backflow occlusion, underscoring the importance of maintaining patent outflow channels throughout the procedure. Future studies incorporating direct ICP monitoring during cervical UBE are needed to establish evidence-based pressure safety thresholds.

The key technical considerations for UBE in the treatment of SSEH are summarized as follows. First, comprehensive preoperative evaluation is crucial for successful surgical outcomes. Preoperative MRI should be performed to identify the responsible segment. Second, establishing precise unilateral biportal access and ensuring adequate visualization are essential. The surgical approach should align with the location of the lesion, selecting either an interlaminar or foraminal approach to facilitate access to the affected segment. Third, during the procedure, the surgeon must remove the ligamentum flavum and hematoma to achieve spinal canal decompression and relieve neural compression. Fine instruments should be used to excise the hematoma, with care taken to avoid injury to the dural sac or nerve roots. Fourth, hemorrhage control using radiofrequency ablation or hemostatic agents is critical for maintaining a clear surgical field. Fifth, a patent outflow cannula should be maintained via a fascial incision oriented perpendicular to the skin incision to prevent an elevation in ICP. Surgeons must be proficient in adjusting irrigation pressure to effectively address these issues. Additionally, intraoperative blood pressure fluctuations may influence bleeding. Therefore, close communication between the surgeon and anesthesiologist is necessary to achieve and maintain an optimal blood pressure within a relatively low and stable range.

## 4 Conclusion

Accurate diagnosis of SSEH requires a comprehensive assessment integrating clinical symptoms and imaging findings to avoid misdiagnosis or underdiagnosis. UBE demonstrates significant advantages in the clinical management of SSEH, including minimal invasiveness, muscle preservation, early recovery, high-definition visualization, and effective spinal decompression. This technique represents a promising and effective therapeutic option for SSEH, particularly for patients prioritizing rapid functional recovery and minimal disruption to daily life. However, further research and long-term follow-up are warranted to validate its efficacy and safety.

## Data Availability

The raw data supporting the conclusions of this article will be made available by the authors, without undue reservation.
